# Applications of soil amendments for enhanced phytostabilization and wheat growth development under combined drought and heavy metal stress

**DOI:** 10.1038/s41598-026-45444-x

**Published:** 2026-04-06

**Authors:** Tianzhi Huang, Imran, Jameel M. Al-Khayri, Mustafa I. Almaghasla

**Affiliations:** 1 Research Center of Rural Environmental Protection and Green Low-Carbon Development, Mianyang Teachers’ College, Sichuan, China; 2 College of Engineering, Agriculture Aviation Innovation Lab, South China Agricultural University, Guangzhou, China; 3 Department of Agricultural Biotechnology, College of Agriculture and Food Sciences, King Faisal University, 31982 Al-Ahsa, Saudi Arabia; 4 Plant Pests and Diseases Unit, College of Agriculture and Food Sciences, King Faisal University, 31982 Al-Ahsa, Saudi Arabia

**Keywords:** Heavy metal, Iron modified nanobiochar, Fe-SZ, Contaminated soil, Yield stability, Environmental sciences, Plant sciences

## Abstract

Heavy metal contamination combined with drought stress severely limits wheat productivity and undermines soil health. This study aimed to evaluate the effectiveness of iron modified stilbite zeolite (Fe-SZ) and nanobiochar (Fe-NB) in improving heavy metal mitigation and drought tolerance in a subsequent wheat crop (*Triticum aestivum* L.) grown in previously contaminated soil. A controlled pot experiment was conducted using three Fe-NB application rates (Fe-NB_1_ = 0%, Fe-NB_2_ = 4%, and Fe-NB_3_ = 8% w/w) and three Fe-SZ particle sizes, (Fe-SZ_1_ = 0.5 mm, Fe-SZ_2_ = 1 mm, and Fe-SZ_3_ = 2 mm), with drought stress imposed 30 days after planting. The most effective treatments significantly improved plant growth and reduced toxic metal uptake. Fe-SZ_2_ decreased arsenic and lead concentrations in plant tissues by 31.33% and 22%, respectively, while Fe-NB_3_ (8% w/w) reduced them by 51.13% and 34%. Cadmium levels were reduced by approximately 50% across Fe-SZ and Fe-NB treatments, and Fe-SZ_2_ further lowered soil Hg by 74.5%. Fe-NB_3_ consistently provided the strongest overall benefits and improvements were reflected in greater shoot biomass, enhanced root growth, and increased chlorophyll content, although some treatments increased metal bioavailability. These findings demonstrate that Fe-SZ and Fe-NB can serve as effective amendments for reducing heavy metal stress and improving wheat resilience under water-limited conditions.

## Introduction

The regulation of plant growth and the remediation of contaminated soils represent major challenges in sustainable agriculture, particularly in environments affected by the dual stress of drought and heavy metal contamination^1^. Drought stress intensifies the adverse impacts of toxic metals such as arsenic (As), lead (Pb), cadmium (Cd), and mercury (Hg) by impairing physiological processes, reducing nutrient availability, and disrupting cellular homeostasis^2^. These metals interfere with root development, oxidative balance, and water uptake, leading to reduced crop productivity and compromised soil health^3^. Both drought and heavy metal contamination arise from natural processes and anthropogenic activities, including industrial emissions, mining, and unsustainable agricultural practices^1,2^. The simultaneous occurrence of these stressors necessitates integrated remediation strategies that improve soil physicochemical properties, enhance water retention, and reduce metal bioavailability.

Organic and inorganic soil amendments have demonstrated considerable potential for addressing these challenges. Nanobiochar (Fe-NB), derived from agricultural biomass and processed to nanoscale dimensions through controlled pyrolysis and ball milling, possesses high surface area and abundant functional groups that facilitate adsorption of heavy metals and improvement of soil structure. Its porous architecture enhances water retention and microbial activity, thereby promoting nutrient cycling and reducing drought-induced stress^3,4^. Similarly, nanozeolite (NZ), produced from natural stilbite zeolite and reduced to nanoscale particles, exhibits strong cation exchange capacity and adsorption properties. These characteristics enable NZ to immobilize metal ions and improve soil fertility and water-holding capacity^5^. However, the remediation efficiency of Fe-NB and NZ can be further enhanced through iron functionalization, which introduces reactive iron species capable of binding and stabilizing toxic metals.

To address this limitation, this study synthesizes iron-modified nanomaterials iron-modified nanobiochar (Fe-NB) and iron-modified nanozeolite (Fe-SZ) using controlled pyrolysis, nanosizing, ion exchange, and chemical co-precipitation techniques. Agricultural biomass (wheat straw) was subjected to slow pyrolysis under oxygen-limited conditions to produce biochar, which was subsequently milled to nanoscale dimensions to enhance surface reactivity (Sect. "Nanobiochar preparation"). Iron functionalization was achieved through adsorption and co-precipitation of iron hydroxides on the nanobiochar surface (Sect. "Iron (Fe) functionalization"), creating reactive sites capable of binding heavy metals and improving soil remediation performance. In parallel, natural stilbite zeolite was nanosized (Sect. "Nanozeolite preparation") and modified via ion exchange and precipitation of iron oxyhydroxide species (Sect. "Iron (Fe) functionalization") to enhance adsorption capacity and structural stability.

The resulting Fe-NB and Fe-NZ combine the advantages of organic and inorganic amendments with the reactivity of iron-based functional groups, enabling superior immobilization of heavy metals and improvement of soil physicochemical properties. Iron modification enhances the sorption of toxic metals through complexation and precipitation mechanisms, reducing their bioavailability and uptake by plants. Additionally, the high surface area and porosity of these materials improve soil moisture retention and microbial activity, fostering a more favorable environment for plant growth under drought stress. Comprehensive characterization (Sect. "Material characterization") confirmed successful nanosizing and iron functionalization, validating their suitability for environmental remediation applications.

Despite growing interest in nanomaterial-based soil amendments, the comparative performance of Fe-NB and Fe-SZ in mitigating combined drought and heavy metal stress remains insufficiently understood. This study therefore evaluates the effectiveness of these iron-modified nanomaterials in improving soil health, reducing metal bioavailability, and enhancing plant growth under drought conditions. By integrating material development with environmental remediation and plant physiological assessment, the research aims to provide a comprehensive framework for sustainable soil management and agricultural productivity in contaminated environments.

## Materials and methods

### Synthesis of iron-modified nanomaterials

#### Nanobiochar preparation

Agricultural biomass (wheat straw) was thoroughly washed with deionized water to eliminate surface impurities and oven-dried at 70 °C for 48 h. Slow pyrolysis was performed in a tubular furnace under oxygen-limited conditions at 550 °C with a heating rate of 10 °C min^−1^ and a abode time of 2 h to achieve complete carbonization. The reactor was allowed to cool naturally to ambient temperature under inert conditions. The resulting biochar was ground and sieved (< 0.5 mm) and subsequently subjected to planetary ball milling at 400 rpm for 8 h to produce nanobiochar. To minimize agglomeration, the milled material was dispersed in deionized water and ultrasonicated at 40 kHz and 300 W for 60 min. The suspension was dried at 60 °C, and particle size distribution was verified using dynamic light scattering (DLS), confirming nanoscale dimensions.

#### Nanozeolite preparation

Natural stilbite zeolite was crushed, washed with deionized water, and oven-dried at 80 °C. The dried material was subjected to planetary ball milling at 350 rpm for 6 h to obtain nanoscale particles. The powder was dispersed in deionized water and ultrasonicated for 30 min to reduce aggregation, followed by drying at 60 °C. Particle size reduction and nanoscale transformation were confirmed through dynamic light scattering (DLS) analysis.

#### Iron (Fe) functionalization

Iron modification of nanobiochar and nanozeolite was achieved using co-precipitation and ion-exchange precipitation methods, respectively. For iron-modified nanobiochar (Fe-NB), a 0.5 M FeCl_3_·6H_2_O solution was prepared with analytical-grade reagents and deionized water. Nanobiochar was suspended in the iron solution at a solid-to-liquid ratio of 1:20 (w/v) and magnetically stirred at 70 °C for 2 h to facilitate Fe^3^_+_ adsorption on the carbon surface. The suspension pH was gradually adjusted to 9.5 using 1 M NaOH under continuous stirring, inducing in situ precipitation of iron hydroxides on the nanobiochar matrix. The mixture was aged for 12 h at room temperature to ensure uniform iron deposition. The solid fraction was separated by centrifugation, repeatedly washed with deionized water until neutral pH was achieved, and oven-dried at 60 °C for 24 h. Iron loading efficiency was quantified by measuring residual iron concentrations in the supernatant using inductively coupled plasma-optical emission spectrometry (ICP-OES).

For iron-modified nanozeolite (Fe-NZ), nanozeolite was suspended in 0.5 M FeCl_3_ solution at a 1:20 (w/v) ratio and stirred at 80 °C for 3 h to promote ion exchange between exchangeable cations (Na_+_ and Ca^2^_+_) and Fe^3^_+_ ions. The pH was subsequently adjusted to 8.5–9.0 using 1 M NaOH to facilitate formation of iron oxyhydroxide species on the zeolite surface. The suspension was aged for 12 h, filtered, and repeatedly washed with deionized water until no free iron was detected in the filtrate. The iron-modified nanozeolite was dried at 70 °C and gently ground prior to storage.

### Material characterization

The synthesized Fe-NB and Fe-SZ were comprehensively characterized to confirm nanosizing and iron functionalization. Surface morphology and particle structure were examined using scanning electron microscopy (SEM) and transmission electron microscopy (TEM). Crystalline phases were identified through X-ray diffraction (XRD), while functional groups were analyzed using Fourier transform infrared spectroscopy (FTIR). Specific surface area and porosity were determined by Brunauer–Emmett–Teller (BET) analysis. Surface charge properties were evaluated via zeta potential and point of zero charge (pHpzc) measurements. Cation exchange capacity (CEC) was determined using the ammonium acetate method. Total iron content was quantified after acid digestion (HNO_3_-HCl mixture) followed by ICP-OES analysis. All synthesis and characterization procedures were conducted in triplicate to ensure reproducibility, and analytical-grade reagents with ultrapure deionized water (18.2 MΩ cm) were used throughout. Glassware was acid-washed prior to use to prevent contamination.

The three particle sizes of Fe-SZ (2, 1 and 0.5 mm) were selected based on our previous findings that indicate smaller particle sizes enhance cation exchange and surface area, improving metal sorption^6,7^ . However, excessively fine particles may reduce soil permeability. Hence, we included a range of particle sizes to evaluate their differential effectiveness under field-relevant conditions. Application rates of 4% and 8% (w/w) for both Fe-SZ and Fe-NB were selected based on prior experiments^7^, which indicated these concentrations optimally improved soil structure, microbial biomass, and metal immobilization. Higher application rates may lead to soil salinization or imbalanced nutrient profiles, whereas lower rates often show marginal effectiveness.

Drought Stress Timing and Duration: Drought was initiated 30 days after planting to allow sufficient vegetative growth, ensuring that plants were physiologically mature enough to respond to water stress. The 60-day experiment duration was chosen to encompass the full stress period and to capture both early and late physiological responses. This approach is consistent with drought simulation studies in pot-based plant trials. The study focused on a drought-sensitive crop species, wheat (*Triticum aestivum* L.), chosen for its significance in agricultural production under drought conditions^6^.The plants were grown from seed, and the treatments were applied at the vegetative stage, which is typically when drought stress has the most significant impact on growth. The soil used in the experiment was loamy soil with low organic matter content to simulate typical drought-prone agricultural soils. The Fe-SZ was mixed thoroughly with the soil before planting. Fe-NB was applied at a concentration of the treatment designed^7^. The Fe-NB was synthesized by pyrolyzing agricultural waste biomass, followed by nanoparticle preparation using a hydrothermal method.

### Drought stress imposition

The plants were subjected to two watering treatments: a control irrigation group receiving optimal water supply (80% field capacity) and a drought stress group, which received reduced water supply (50% field capacity) for 14 days. The drought stress treatment was initiated after 30 days of plant growth when the plants had reached the vegetative stage. The drought treatment continued until the end of the experiment, which lasted for 60 days.

### Soil physicochemical characterization and analytical methods

The soils used in this experiment were sourced from a technogenically contaminated site and were analyzed for their physicochemical properties using standard methods. The cation exchange capacity (CEC) was determined using the ammonium acetate method at pH 7, while organic carbon (OC) content was measured using the Walkley–Black wet oxidation technique. Total nitrogen (TN) was analyzed through the Kjeldahl digestion method, and the carbon-to-nitrogen (C/N) ratio was calculated by dividing OC by TN. Available phosphorus (Olsen P) was extracted using the Olsen method, which employs sodium bicarbonate, ideal for soils with neutral to alkaline pH. Soil pH was measured in a 1:2.5 soil-to-water suspension using a pH meter, and electrical conductivity (ECe) was determined using a conductivity meter to assess soil salinity. Bulk density was measured by the core method, involving the drying of a known volume of soil to calculate the weight per unit volume. Finally, soil texture, including the proportion of sand, silt, and clay, was determined using the hydrometer method.

### Soil characterization

The experimental soil was collected from the plow layer (0–20 cm depth) and characterized prior to the establishment of the pot experiment. Analysis revealed a sandy loam texture, comprising 52.5% sand, 28.0% silt, and 19.5% clay, as per the USDA soil textural classification. The soil was slightly acidic, with a pH (H_2_O) of 5.30, and exhibited low salinity (ECe = 0.22 dS m^−1^). Fertility status was notably low, as indicated by a moderate cation exchange capacity (CEC) of 29.0 Cmol_+_ kg^−1^ soil, coupled with deficient levels of organic carbon (OC = 1.00%), total nitrogen (TN = 0.04%), and available phosphorus (Olsen *P* = 1.50 mg kg^−1^). The resultant carbon-to-nitrogen (C/N) ratio of 25 suggests a potential for nitrogen immobilization during decomposition of organic amendments. The soil’s bulk density was 1.35 g cm^−3^, which is within a typical range for agricultural soils. This baseline characterization confirms a substrate with inherently low fertility and limited nutrient-holding capacity, providing a relevant model system for evaluating the efficacy of soil amendments aimed at enhancing productivity and stress resilience.

### Experimental design

The experiment was conducted as a three-factor factorial arrangement in a Completely Randomized Design (CRD) under greenhouse conditions. The factors included: stilbite zeolite (Fe-SZ) particle size (three levels: 0.5, 1, and 2 mm), Fe-SZ application rate (0, 4, and 8% w/w), and Fe-nanobiochar (Fe-NB) application rate (0, 4, and 8% w/w). The combination of these factors resulted in 27 treatment combinations (3 × 3 × 3), each replicated three times, yielding 81 experimental units (Table 1). Each pot contained contaminated soil amended according to its assigned treatment. Pots were randomly arranged and periodically repositioned within the greenhouse to minimize positional effects. Drought stress was imposed 30 days after planting to evaluate amendment performance under water-limited conditions. Throughout the experimental period, all pots received identical irrigation and environmental conditions to reduce variability.

**Table 1:** Analysis of variance (ANOVA) for heavy metal concentrations in plant tissue.

Source of Variation	df
SZ Particle Size (A)	2
SZ Rate (B)	2
NBC Rate (C)	2
A × B	4
A × C	4
B × C	4
A × B × C	8
Error	54
Total	80

These seven combinations allowed evaluation of both particle size and application rate effects in an integrated manner while maintaining experimental simplicity and interpretability. Pots were randomly arranged and periodically repositioned to minimize positional effects. Drought stress was imposed 30 days after planting to simulate water-limited conditions and evaluate amendment performance under combined stress. Each experimental unit consisted of one pot containing contaminated soil amended according to treatment specification. A pot experiments were conducted in the greenhouse, examining three factors: particle sizes of Fe-SZ (2 mm for coarse-grained, 1 mm for medium-grained, and 0.5 mm for fine-grained), Fe-SZ application rates (0%, 4%, and 8% w/w), and Fe-NB application rates (0%, 4%, and 8% w/w) and its chemical properties are presented in Table 2. Pots were randomly arranged on greenhouse benches to eliminate positional bias. To minimize environmental variation within the greenhouse, pots were repositioned weekly. All pots were maintained under identical irrigation and environmental conditions throughout the experimental period.

**Table 2:** Selected properties of the Fe-SZ and Fe-NB used for the study.

Treatments properties	Fe-SZ		Properties	Fe-NB	
	Unit	Quantity		Unit	Quantity
CEC	C mol kg^−1^ clay	136	CEC	C mol kg^−1^ clay	20
SiO_2_	%	62.80	OC	%	10.50
Al_2_O_3_	%	8.90	TN	%	1.80
CaO	%	7.40	C/N		5.83
P_2_O_5_	%	0.22	MC	%	45.12
K_2_O	%	0.04	Olsen P	mg kg^−1^	4.50
Fe_2_O_3_	%	0.02	pH-H_2_O		7.80
TiO_2_	%	0.011	ECe	(dSm^−1^)	0.25
Loss on ignition	%	14	S	mg g^−1^	1.02
Specific surface area	m^2^g^−1^	8.8			

### Agronomic practices

This study presents an innovative approach to mitigate heavy metal, improve soil productivity and optimize wheat cultivation under contaminated low carbon soil, Fe-SZ samples were processed into three distinct particle size fractions (0.5, 1, and 2 mm) using a disc mill and sieving. These fractions were then applied at varying rates (0, 4, and 8% w/w) to investigate their effects on wheat growth and productivity. Additionally, Fe-NB was incorporated at rates of 0, 4, and 8% w/w to enhance soil fertility and mitigate the heavy metal and facilitate plant growth and development. After a 10-day equilibration period, 10 wheat seeds were planted pot^−1^, and thinned to 3–5 seedlings pot^−1^ after emergence. Soil moisture (irrigation) was maintained at approximately 80% field capacity throughout the experiment. Irrigation was provided regularly to ensure adequate water availability for the plants, particularly during critical growth stages. Fertilization was applied uniformly across all treatments using a balanced N-P-K fertilizer at concentrations of 300 ppm N, 225 ppm P, and 150 ppm K, ensuring sufficient nutrient availability for optimal growth. The total aboveground biomass of wheat was harvested at maturity to determine grain yield and yield components.

### Statistical analysis

Data were analyzed using three-way analysis of variance (ANOVA) appropriate for a factorial experiment in a Completely Randomized Design. The statistical model included the main effects of SZ particle size (A), SZ rate (B), and NBC rate (C), as well as their interaction effects (A × B, A × C, B × C, and A × B × C). Treatment means were compared using the Least Significant Difference (LSD) test at P ≤ 0.05 when F-tests were significant^8^. Furthermore, regression analysis was performed using R^9^. Fe-SZ particle size and rate; and Fe-NB rate and their interaction means were separated at *P* ≤ 0.05 by Fisher’s least significant difference (LSD) test.

## Results

### Factorial effects on heavy metal uptake

The three-way ANOVA revealed that Fe-SZ particle size, Fe-SZ application rate, and Fe-NB rate significantly influenced heavy metal concentrations in plant tissues (*P* ≤ 0.05). Significant interaction effects among factors were observed for several metals, indicating that the response to one amendment depended on the level of the other factors. Therefore, treatment effects were interpreted considering both main and interaction effects. The combined application of Fe-SZ and Fe-NB generally reduced heavy metal bioavailability more effectively than individual applications, confirming a synergistic interaction between amendments.

### Drought stress

**Fig. 1:**
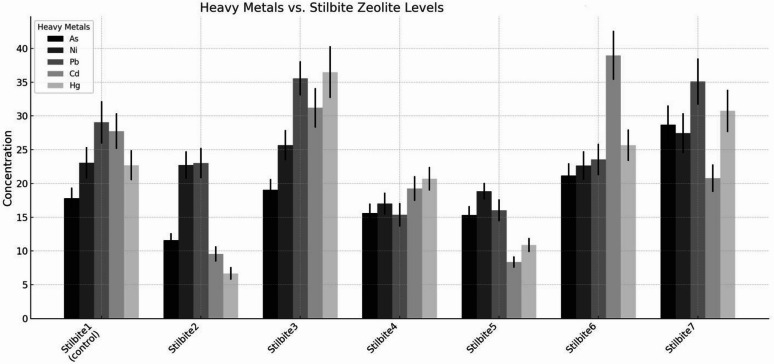
Effects of stilbite zeolite on heavy metal concentrations included: Fe-SZ1, 0 rate (control), Fe-SZ2, fine-grained (0.5 mm) at 4 ton ha^−1^, Fe-SZ3, medium-grained (1 mm) at 4% w/w, Fe-SZ4, coarse-grained (2 mm) at 4% w/w, Fe-SZ5, fine-grained (0.5 mm) at 8% w/w, Fe-SZ6, medium-grained (1 mm) at 8% w/w, and Fe-SZ7, coarse-grained (2 mm) at 8% w/w. Bars represent means with standard deviations (± SD). Distinct greyscale hatch patterns differentiate heavy metals for clear visualization in monochrome reproduction.

**Fig. 2:**
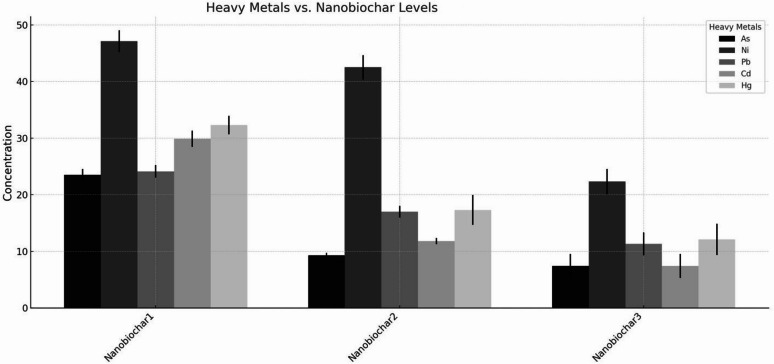
Effects of nanobiochar treatments on heavy metal included: Fe-NB1, 0 rate, Fe-NB2, 4% w/w, and Fe-NB3, 8% w/w. Bars represent means with standard deviations (± SD). Distinct greyscale hatch patterns differentiate heavy metals for clear visualization in monochrome reproduction.

The drought-stressed control (Fe-SZ_1_) exhibited reduced agronomic performance, with plant height, spike length, and grain yield recorded at 78.3 ± 3.11 cm, 13.2 ± 1.14 cm, and 1152 kg ha^−1^, respectively (Fig. 1). Application of Fe-SZ at 4% w/w (Fe-SZ_2_) resulted in higher values for these parameters, with plant height of 83.2 ± 2.91 cm, spike length of 14.3 ± 1.23 cm, and grain yield of 1357 kg ha^−1^. At 8% w/w (Fe-SZ_3_), plant height reached 80.5 ± 2.88 cm, spike length 14.1 ± 1.12 cm, and grain yield 1320 kg ha^−1^. The 8% (w/w) Fe-SZ treatment (Fe-SZ_4_) recorded the highest values among Fe-SZ applications, with plant height of 86.1 ± 2.74 cm, spike length of 15.1 ± 1.34 cm, and grain yield of 1493 kg ha^−1^. Corresponding Fe-NB results at the 4% w/w rate (Fe-NB_1_), plant height, spike length, and grain yield were 79.5 ± 3.12 cm, 13.4 ± 1.08 cm, and 1187 kg ha^−1^, respectively (Fig. 2). The 8% w/w Fe-NB treatment (Fe-NB_**2**_) showed higher values, with plant height of 85.3 ± 2.99 cm, spike length of 15.4 ± 1.16 cm, and grain yield of 3421 kg ha^−1^. The maximum values across all Fe-NB treatments were observed at 8% w/w Fe-NB_3_, where plant height reached 88.9 ± 3.02 cm, spike length 15.9 ± 1.21 cm, and grain yield 3576 kg ha^−1^.

### Leaf water potential and drought stress alleviation

The combined application of Fe-SZ and Fe-NB at their optimal rates (8% w/w, Fe-SZ_3_ + 8% w/w, Fe-NB_3_) demonstrated a synergistic effect in mitigating drought stress, resulting in superior agronomic performance (Table 3). This treatment significantly enhanced plant height to 91.2 ± 2.91 cm (a 16.5% increase over the control), spike length to 16.2 ± 1.19 cm, and grain yield to 3682 kg ha^−1^, representing a substantial 45.9% increase compared to the drought-stressed control. The combined Fe-SZ3 + Fe-NB3 treatment markedly improved leaf water potential (ΨL), increasing it to -0.72 ± 0.05 MPa from a control value of -1.15 ± 0.07 MPa, indicating superior water uptake and retention. The drought-induced reduction in relative water content (RWC) was effectively alleviated, with RWC rising to 78.3 ± 2.91% compared to 68.5 ± 3.29% in the control. Stomatal conductance (gₛ) also improved significantly, reaching 112.3 ± 6.21 mmol m^−2^ s^−1^ under the Fe-SZ3 + Fe-NB3 treatment, in contrast to the severely reduced 90.1 ± 5.31 mmol m^−2^ s^−1^ in the control.

**Table 3:** Effects of combined application of Iron modified Stilbite Zeolite (Fe-SZ_3_) and Iron modified Nanobiochar (Fe-NB_3_) on plant growth, physiological traits, and water relations of drought stressed plants.

Factors	Plant Height (cm)	Spike Length (cm)	Grain Yield (kg ha^−1^)	Leaf Water Potential (MPa)
Control (No Fe-SZ/Fe-NB)	78.3 ± 2.91	14.5 ± 1.19	2525 ± 120	− 1.15 ± 0.07
Fe-SZ_3_ + Fe-NB_3_ (8% w/w each)	91.2 ± 2.91	16.2 ± 1.19	3682 ± 150	− 0.72 ± 0.05
LSD 0.05	3.2	1.5	180	0.08
SEM	1.2	0.5	60	0.03

Values represent mean ± standard deviation (SD). LSD and SEM indicate the least significant difference at *p* ≤ 0.05 and standard error of the mean, respectively.

### Water use efficiency (WUE)

Under drought stress conditions, soil moisture content was significantly higher in the Fe-SZ and Fe-NB-treated plots compared to the control. In the control group, soil moisture was 12.3 ± 1.29%, while Fe-SZ_3_ + Fe-NB_3_ treatments increased soil moisture content to 17.6 ± 1.43%, demonstrating enhanced soil water retention capacity. Fe-SZ and Fe-NB treatments significantly improved WUE under drought stress. The control group had a WUE of 3.4 ± 0.25 kg m^−3^, while Fe-SZ_3_ + Fe-NB_3_ treatment improved WUE to 4.7 ± 0.32 kg m^−3^, indicating that these treatments allow for more efficient use of water during drought conditions, leading to better plant performance and higher yields (Table 4).

**Table 4:** Effects of combined application of Stilbite Zeolite (Fe-SZ3) and Nanobiochar (Fe-NB3) on RWC %, Stomatal conductance and water relations of soil and plant under drought stressed.

Factors	RWC (%)	Stomatal Conductance (mmol m^−2^ s^−1^)	Soil Moisture (%)	WUE (kg m^−3^)
Control (No Fe-SZ/Fe-NB)	68.5 ± 3.29	90.1 ± 5.31	12.3 ± 1.29	3.4 ± 0.25
Fe-SZ_3_ + Fe-NB3 (8% w/w each)	78.3 ± 2.91	112.3 ± 6.21	17.6 ± 1.43	4.7 ± 0.32
LSD 0.05	3.0	6.0	1.4	0.3
SEM	1.2	2.1	0.5	0.1

Values represent mean ± standard deviation (SD). LSD and SEM indicate the least significant difference at p ≤ 0.05 and standard error of the mean, respectively.

### Particle size and application rate (% w/w) of Fe-SZ and heavy metal mitigation

**Fig. 3:**
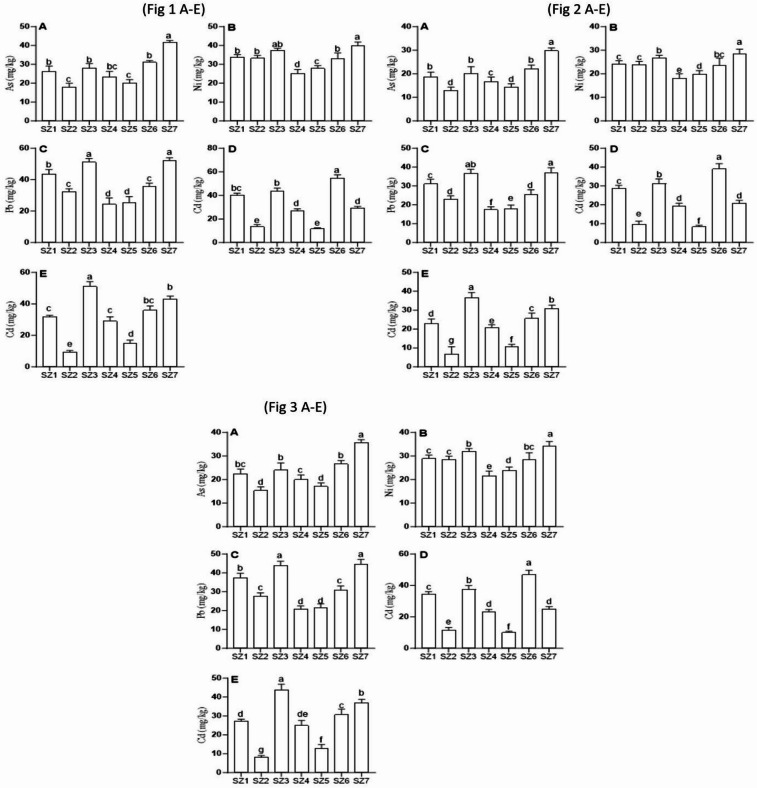
Fe-SZ and particle size and level impact on heavy metal mitigation (1 A-E), Heavy metal mitigation with 8% w/w, Fe-NB in combination with Fe-SZ (2 A-E) and Heavy metal mitigation with 4% w/w, Fe-NB application in combination with Fe-SZ (3 A-E).

The application of Fe-SZ amendments significantly influenced the bioavailability and accumulation of heavy metals in wheat grown under drought stress (Table 4). Relative to the unamended control (Fe-SZ_1_), the treatments exhibited distinct, rate-dependent effects on the concentrations of arsenic (As), nickel (Ni), lead (Pb), cadmium (Cd), and mercury (Hg). The Fe-SZ_2_ and Fe-SZ_5_ treatments demonstrated the highest efficacy in reducing metal uptake (Fig. 3). Fe-SZ_2_ application resulted in pronounced decreases, particularly for Cd and Hg, which were reduced by 65.6% and 69.1%, respectively, compared to the control. Similarly, Fe-SZ_5_ led to a 69.7% reduction in Cd and a 50.9% reduction in Hg, indicating a strong immobilization capacity for these elements (Fig. 1). The Fe-SZ_4_ treatment also showed a moderate remediation potential, notably reducing Ni and Pb concentrations by 25.1% and 43.4%, respectively. The Fe-SZ_6_ treatment produced a mixed profile, with As and Ni levels comparable to the control, but a substantial spike in Cd concentration to 39.48 ± 3.63 mg kg^−1^. The Fe-SZ_7_ treatment resulted in the most severe exacerbation, significantly elevating the concentrations of all analyzed metals, indicating a complete loss of remediation effectiveness and a potential for exacerbating metal toxicity.

### Particle size application rate (% w/w) of Fe-NB and heavy metal mitigation

The application of Fe-NB amendments significantly altered the bioavailability and accumulation of heavy metals in wheat, with efficacy being strongly dependent on the application rate (Table 5). The Fe-NB_1_ treatment exhibited a variable metal immobilization capacity. While it resulted in concentrations of As (24.51 ± 1.02 mg kg^−1^), Pb (25.13 ± 1.13 mg kg^−1^), and Cd (29.91 ± 1.43 mg kg^−1^) that were lower than the control, it concurrently led to relatively high concentrations of Ni (48.11 ± 1.93 mg kg^−1^) and Hg (32.31 ± 1.63 mg kg^−1^). This treatment significantly decreased the concentrations of all metals, with particularly notable reductions for As (10.31 ± 0.43 mg kg^−1^, a 55.8% decrease from Fe-NB_1_) and Pb (18.01 ± 1.03 mg kg^−1^, a 42.1% decrease from Fe-NB1). The most effective remediation was observed with the NC3 treatment, which yielded the lowest concentrations for all metals analyzed: As (8.41 ± 2.13 mg kg^−1^), Ni (23.31 ± 2.23 mg kg^−1^), Pb (12.31 ± 2.03 mg kg^−1^), Cd (7.41 ± 2.13 mg kg^−1^), and Hg (12.11 ± 2.75 mg kg^−1^).

**Table 5:** Effect of Fe-SZ particle size, rate and Fe-NB rate on the yield and yield component of wheat.

Treatments	Height (cm)	spike length (cm)	Grain spike^−1^	Grain yield (g pot^−1^)	Converted Grain yield (kg ha^−1^)	Biomass yield (g pot^−1^)	Converted Biomass yield (kg ha^−1^)
Fe-SZ rate and particle size							
No Fe-SZ_1_ (control)	73.23c	11.73c	22.23c	12.80e	2394.1e	20.91a	3525.6a
Fe-SZ_2_	91.43a	14.63a	25.73a	15.89c	1719.1b	22.24a	3882.1a
Fe-SZ_3_	87.03bc	14.33b	24.23b	16.55b	1619.3c	21.55a	3677.0a
Fe-SZ_4_	85.53c	14.03a	22.03c	14.96de	1439.4de	21.26a	3600.3a
Fe-SZ_5_	91.63a	14.83a	26.73a	17.41a	1874.9a	22.74a	4073.7a
Fe-SZ_6_	88.93ab	14.13b	24.23b	16.79b	1688.0b	22.16a	3900.9a
Fe-SZ_7_	89.03ab	14.13b	22.93bc	14.07d	1473.9d	22.04a	3852.8a
LSD_0.05_	2.2	11.1	1.5	0.33	55.6	42.54	889.22
Nanobiochar (Fe-NB) rate (% w/w)							
Fe-NB_1_	84.549c	12.73c	21.83c	13.33c	1229.14c	21.16a	3592.2a
Fe-NB_2_	89.073b	13.03b	24.63b	14.55b	1587.75b	21.95a	3836.7a
Fe-NB_3_	91.74a	14.33a	28.84a	15.69a	1989.94a	22.41a	3950.9a
LSD_0.05_	2.1388	0.15	0.9	0.15	35.162	0.1487	582.13
Overall	79.52	14.94	26.14	14.9	1669.3	4.93	3871.6
SEM	0.65	0.54	0.46	0.54	45.90	0.54	110.6

Means along the same column with different superscripts are significantly different, CV-Coefficient Of Variation, SEM standard error of the mean, Fe-SZ_1_ 0 rate Fe-SZ (control), Fe-SZ_2_—fine-grained (0.5 mm) Fe-SZ at 4 ton/ha, Fe-SZ_3_—medium-grained (1 mm) Fe-SZ at 4% w/w, Fe-SZ_4_—coarse-grained (2 mm) Fe-SZ at 4% w/w, Fe-SZ_5_—fine-grained (0.5 mm) Fe-SZ at 8% w/w, Fe-SZ_6,_ medium-grained (1 mm) Fe-SZ at 8% w/w, Fe-SZ_7_—coarse-grained (2 mm) Fe-SZ at 8% w/w, Fe-NB_1_—0 rate, Fe-NB_2_—4% w/w rate and Fe-NB_3_—8% w/w rate.

### Yield and yield component response

The agronomic parameters of wheat were differentially influenced by the application rate and particle size of Fe-SZ and Fe-NB soil amendments under drought conditions (Table 4). A maximum height increase of 8% w/w, was observed with the application of 0.5 mm Fe-SZ at 4% and 8% rates, as well as with 8% w/w Fe-NB. The application of fine-grained (0.5 mm) amendments was particularly effective. Spike length increased from 13.87 cm in the control to 14.74 cm and 14.94 cm with 4% w/w and 8% w/w Fe-SZ, respectively, and to 15.38 cm with 8% w/w Fe-NB. The highest values for grains per spike (28.89 and 29.90, respectively) were recorded with the 8% w/w application of 0.5 mm Fe-SZ and Fe-NB. The synergistic interaction between the amendments was pronounced. The co-application of 0.5 mm Fe-SZ and Fe-NB, both at 8% w/w, produced the greatest improvements, resulting in a spike length of 15.83 cm (control: 13.40 cm) and 34.33 grains per spike (control: 22.33). This treatment combination also maximized grain yield, achieving 2544 kg ha^−1^, a substantial increase over the control yield of 1057 kg ha^−1^. Wheat yield showed an increasing trend with increasing soil nutrient status. Much of the yield increase (91%) was explained by the improvement in soil OC while the second most important factor was AP, which explained 83% of the variation in yield.

### Uptake and tissue concentration of heavy metal

Statistical analysis confirmed highly significant differences in heavy metal concentrations across the various Fe-SZ and Fe-NB amendments, underscoring their potential as sustainable strategies for mitigating metal uptake in wheat under combined drought and contamination stress (Table 5). The efficacy of both Fe-SZ and Fe-NB amendments was highly treatment-specific, with optimal application rates leading to significant metal immobilization, while suboptimal rates occasionally increased bioavailability. Arsenic (As) Dynamics: Relative to the control (Fe-SZ_1_: 0.18 ± 0.05 mg kg^−1^), As concentration was most effectively reduced by the Fe-NB_3_ treatment (0.10 ± 0.04 mg kg^−1^; 44.44% decrease), followed by Fe-NB_2_ and Fe-SZ_5_. In contrast, several treatments exacerbated As accumulation, with the highest increase observed in Fe-SZ_7_ (0.28 ± 0.05 mg kg^−1^; 55.56% increase), indicating a potential adverse effect at that specific application rate. Nickel (Ni) and Lead (Pb) Response: For Ni, the most substantial reduction from the control (1.23 ± 0.13 mg kg^−1^) was achieved with Fe-SZ_4_ (1.13 ± 0.12 mg kg^−1^) and Fe-NB3 (1.13 ± 0.12 mg kg^−1^). Fe-NB treatments except Fe-NB_3_ increased Ni levels, with Fe-NB_1_ showing a 16.26% rise. A similar pattern was observed for Pb (control: 0.13 ± 0.04 mg kg^−1^), where Fe-SZ_2_ and Fe-SZ_5_ were the most effective, reducing concentrations to 0.11 ± 0.04 mg kg^−1^ (15.38% decrease). The Fe-SZ_3_ treatment, however, increased Pb by 15.38%.

However, Fe-SZ_7_ raised Pb levels to 0.16 ± 0.04 mg kg^−1^ (23.08% increase). Fe-NB_1_ increased Pb to 0.14 ± 0.04 mg kg^−1^ (7.69%), Fe-NB2 maintained 0.11 ± 0.04 mg kg^−1^, and Fe-NB3 decreased Pb further to 0.10 ± 0.04 mg kg^−1^ (23.08% reduction). For Cd, Fe-SZ_1_ had 0.05 ± 0.031 mg kg^−1^. Fe-SZ_2_ and Fe-SZ_5_ both decreased Cd to 0.04 ± 0.031 mg kg^−1^ (20% decrease), while Fe-SZ_3_ raised it to 0.06 ± 0.032 mg kg^−1^ (20% increase). Fe-SZ_4_ reduced Cd slightly to 0.045 ± 0.031 mg kg^−1^ (10%), Fe-SZ6 further increased it to 0.065 ± 0.033 mg kg^−1^ (30%), and Fe-SZ_7_ showed no change (0.05 ± 0.031 mg kg^−1^). Fe-NB_1_ increased Cd to 0.055 ± 0.031 mg kg^−1^ (10%), Fe-NB_2_ lowered it to 0.045 ± 0.031 mg kg^−1^ (10%), and Fe-NB3 maintained the lowest value at 0.04 ± 0.031 mg kg^−1^ (20% decrease). Mercury (Hg) levels in Fe-SZ_1_ were 0.04 ± 0.031 mg kg^−1^. Fe-SZ_2_ brought a substantial decline to 0.035 ± 0.0305 mg kg^−1^ (12.5% decrease), while Fe-SZ_3_ and Fe-SZ_7_ increased Hg to 0.045 ± 0.0315 mg kg^−1^ (12.5% increase). Fe-SZ_4_ remained unchanged at 0.04 ± 0.031 mg kg^−1^, Fe-SZ_5_ decreased Hg slightly to 0.037 ± 0.0307 mg kg^−1^, and Fe-SZ_6_ increased it to 0.042 ± 0.0312 mg kg^−1^. Fe-NB_1_ showed the most pronounced increase at 0.05 ± 0.0315 mg kg^−1^ (25%), while Fe-NB_2_ stayed at 0.04 ± 0.031 mg kg^−1^, and Fe-NB_3_ brought it down to 0.038 ± 0.0308 mg kg^−1^ (5%).

### Association among heavy metals, wheat agronomic traits, and stilbite zeolite application

The correlation analysis among heavy metals, wheat growth, and yield parameters under Fe-SZ amendment provides critical insights into the complex interactions governing plant performance under stress. The relationships between metal concentrations and plant traits were multifaceted. Notably, a positive correlation was observed in some cases between trace amounts of arsenic (As) or cadmium (Cd) and plant biomass. The immobilization of heavy metals by Fe-SZ, thereby reducing their bioavailability, was reflected in the attenuated negative correlations between metal concentrations and plant growth traits in treated soils compared to untreated controls. This indicates a successful reduction of metal-induced stress.

### Association among heavy metals, wheat agronomic traits, and nanobiochar application

The relationships between heavy metal concentrations and key agronomic traits in wheat, as visualized by a correlogram, provide critical insights into the impact of contamination on crop productivity. The analysis revealed two dominant patterns. First, strong positive correlations were observed among the heavy metals themselves, particularly between Pb–Cd (r = 0.80), As-Ni (r = 0.79), As-Cd (r = 0.79), and Cd-Hg (r = 0.80). This suggests a co-contamination effect, where the presence of one metal is often associated with others. Second, these metals collectively exhibited a consistent negative correlation with plant growth and yield. All agronomic traits plant height, spike length, grains per spike, grain yield, and biomass yield were negatively correlated with the measured metals. The detrimental effect was most pronounced on yield components, with strong negative correlations observed between the metals and grains per spike (e.g., Cd, r = -0.48), grain yield, and biomass yield. Conversely, the agronomic traits showed strong positive intercorrelations. Plant height was highly correlated with grains per spike (r = 0.92) and biomass yield (r = 0.96), while grains per spike showed a near-perfect positive correlation with grain yield (r = 0.98).

### Synergistic effect of Fe-NB and Fe-SZ on soil fertility and nutrients concentration

The initial characterization of the soil amendments revealed distinct properties for stilbite-zeolite (Fe-SZ) and nano-biochar (Fe-NB). The Fe-SZ exhibited a high cation exchange capacity (CEC) and was rich in calcium, with a mineral composition dominated by SiO_2_ (63%), followed by Al_2_O_3_ (8.9%) and CaO (7.4%). In contrast, the Fe-NB possessed a lower CEC (20 cmol_+_ kg^−1^) and a C/N ratio of 5.83. The application of these amendments differentially influenced key soil properties (Table 6). Soil pH was significantly affected (*p* < 0.05) by the rate and particle size of Fe-SZ, but not by Fe-NB application or the Fe-SZ × Fe-NB interaction. The highest pH value relative to the control was achieved with the application of 0.5 mm Fe-SZ at an 8% w/w rate. Conversely, soil electrical conductivity (ECe) was significantly affected (*p* < 0.05) only by Fe-NB application, showing an increasing trend, though it was not significantly influenced by Fe-SZ or the interaction effect. Soil CEC was significantly affected (*p* < 0.05) by all factors: Fe-SZ rate, Fe-SZ particle size, Fe-NB rate, and their interaction. The most substantial increases in CEC were observed with the co-application of fine-grained (0.5 mm) amendments. Applications of 0.5 mm Fe-SZ at 4% w/w and 8% w/w, increased CEC by 14.95% and 16.08%, respectively, while 8% w/w, Fe-NB alone increased CEC by 20.04%. The highest CEC values were recorded when both fine-grained Fe-SZ and Fe-NB were applied at 8% w/w.

**Table 6:** Heavy metal concentration (mg kg^−1^) in wheat grain after harvest.

Factors	As	Ni	Pb	Cd	Hg
Fe-SZ rate and particle size					
NoFe-SZ_1_ (Control)	0.13 ± 0.01	1.16 ± 0.09	0.06 ± 0.01	0.012 ± 0.002	0.021 ± 0.002
Fe-SZ_2_	0.09 ± 0.02	1.12 ± 0.08	0.05 ± 0.01	0.010 ± 0.002	0.019 ± 0.001
Fe-SZ_3_	0.11 ± 0.02	1.15 ± 0.07	0.06 ± 0.01	0.009 ± 0.001	0.018 ± 0.002
Fe-SZ_4_	0.10 ± 0.01	1.09 ± 0.08	0.05 ± 0.01	0.008 ± 0.001	0.017 ± 0.001
Fe-SZ_5_	0.09 ± 0.02	1.11 ± 0.09	0.05 ± 0.01	0.009 ± 0.002	0.018 ± 0.001
Fe-SZ_6_	0.08 ± 0.01	1.05 ± 0.07	0.04 ± 0.01	0.007 ± 0.001	0.016 ± 0.001
Fe-SZ_7_	0.07 ± 0.01	1.02 ± 0.06	0.04 ± 0.01	0.006 ± 0.001	0.015 ± 0.001
LSD 0.05	3.2671	3.1054	1.5066	3.2671	3.1054
Fe-Nanobiochar rate (% w/w)					
Fe-NB_1_	0.15 ± 0.03	1.27 ± 0.11	0.08 ± 0.02	0.00 ± 0.001	0.02 ± 0.001
Fe-NB_2_	0.08 ± 0.02	1.13 ± 0.08	0.06 ± 0.01	0.00 ± 0.001	0.025 ± 0.0005
Fe-NB_3_	0.16 ± 0.03	1.24 ± 0.09	0.10 ± 0.03	0.01 ± 0.002	0.015 ± 0.0015
LSD0.05	0.014	0.16	0.008	0.0015	0.001
SEM	0.18 ± 0.01	1.28 ± 0.08	0.06 ± 0.01	0.01 ± 0.001	0.015 ± 0.0015

### Soil nutrients and total organic carbon (TOC)

The application of Fe-SZ and Fe-NB amendments significantly enhanced key soil fertility parameters, with the most pronounced improvements observed at the highest application rates of fine-grained materials (Table 7). Total organic carbon (TOC) was significantly increased (*p* < 0.05) by the individual factors of Fe-SZ rate, Fe-SZ particle size, and Fe-NB rate, though their interaction was not significant. The co-application of 0.5 mm Fe-SZ and Fe-NB, each at 8% w/w, was most effective, increasing TOC by 23.74% and 11.03%, respectively, over the control. Similarly, total nitrogen content was significantly affected by Fe-SZ and Fe-NB individually. Application of 0.5 mm Fe-SZ at 8% w/w and Fe-NB at 8% w/w increased soil nitrogen by 14.29% and 19.05%, respectively. Available phosphorus (AP) was the most responsive parameter, being significantly influenced by all factors, including the Fe-SZ × Fe-NB interaction. The 8% w/w application of 0.5 mm Fe-SZ increased AP by 33.79%, while the same rate of Fe-NB led to a dramatic 328% increase. A strong synergistic effect on both cation exchange capacity (CEC) and AP was confirmed. The co-application of 0.5 mm Fe-SZ and Fe-NB at 8% w/w, yielded the highest values for CEC (52.64 cmol_+_ kg^−1^) and AP (17.45 mg kg^−1^), substantially greater than the control (CEC: 31.42 cmol_+_ kg^−1^; AP: 4.07 mg kg^−1^).

**Table 7:** Effect of Fe-SZ particle size and rate and Fe-NB rate on soil chemical properties of the growing media.

Factors	Chemical soil properties					
	pH_H2O_	ECe (dS/m)	OC (%)	TN (%)	CEC (Cmol ( +) kg^−1^ soil)	Olsen P (mg kg^−1^)
Fe-SZ rate and particle size						
No Fe-SZ_1_ (Control)	6.51c	0.74a	0.38f.	0.86d	40.28e	7.63d
Fe-SZ_2_	6.78ab	0.73a	0.50b	0.84ba	46.46ba	9.09cb
Fe-SZ_3_	6.71bc	0.73a	0.47 cd	0.84bdac	45.14bc	9.06cb
Fe-SZ_4_	6.64bc	0.74a	0.41ef	0.85dc	41.96d	8.26 cd
Fe-SZ_5_	6.95a	0.69a	0.54a	0.83a	46.94a	10.57a
Fe-SZ_6_	6.74ab	0.72a	0.49cb	0.84bac	45.86bac	9.64b
Fe-SZ_7_	6.66bc	0.73a	0.44ed	0.85bdc	44.58c	9.06cb
LSD_0.05_	0.233	0.105	0.0335	0.0115	1.4244	0.909
Fe-Nanobiochar rate (% w/w)						
Fe-NB_1_	6.68a	0.64a	0.32c	0.86c	40.91c	4.30c
Fe-NB_2_	6.74a	0.75b	0.47b	0.84b	45.26b	9.42b
Fe-NB_3_	6.79a	0.79b	0.65a	0.82a	49.31a	13.99a
LSD_0.05_	0.17	0.06	0.02	0.01	1.02	0.67
Overall	7.78	0.34	1.53	0.23	45.53	10.07
SEM	0.034	0.014	0.018	0.002	0.636	0.523

The means along the same column with different superscripts are significantly different, CV—coefficient of variation, SEM—standard error of the mean, Fe-SZ_1—_0 rate Fe-SZ (control), Fe-SZ_2_—fine grained (0.5 mm) Fe-SZ at 4 ton/ha, Fe-SZ_3_—medium-grained (1 mm) Fe-SZ at 4% w/w, Fe-SZ_4,_ coarse-grained (2 mm) Fe-SZ at 4% w/w, Fe-SZ_5_—fine-grained (0.5 mm) Fe-SZ at 8% w/w, Fe-SZ_6_ , medium-grained (1 mm) Fe-SZ at 8% w/w , Fe-SZ_7_—coarse-grained (2 mm) Fe-SZ at 8% w/w , Fe-NB_1_ 0, Fe-NB_2_—4% w/w and Fe-NB_3_—8% w/w.

## Discussions

The present study demonstrates that combined application of stilbite-zeolite (Fe-SZ) and nano-biochar (Fe-NB) effectively mitigates two critical abiotic stresses heavy metal (HM) toxicity and drought in wheat. The significant reduction in shoot arsenic (As) and lead (Pb) concentrations, coupled with enhanced physiological parameters and biomass under water deficit in treatments Fe-SZ2 and Fe-NB3, robustly supports our hypothesis. These results align with the growing recognition of mineral- and carbon-based amendments as multifunctional soil conditioners^7,10^. The following sections provide a critical synthesis of these findings by delineating the proposed mechanistic framework, situating the results within the extant literature, reconciling paradoxical observations, and explicitly addressing the methodological scope of this work.

### Synergistic mechanisms of Fe-SZ and Fe-NB of stress amelioration

The dual benefit observed likely stems from complementary physiochemical and putative physiological pathways. HM immobilization is primarily governed by the high cation exchange capacity (CEC) and porous structure of Fe-SZ, which sequesters metal ions via ion exchange, surface complexation, and precipitation of insoluble metal-silicate compounds^11,12^. Concurrently, Fe-NB contributes through its nanoscale-enabled high specific surface area and abundant oxygen-functional groups (–COOH, –OH), facilitating chemisorption and electrostatic binding of metal cations^13,14^. The synergy between these materials promotes the formation of stable metal complexes, reducing the phytoavailable pool in soil solution^13^.

Drought resilience is enhanced through improved soil hydro-physical properties. Fe-SZ acts as a microporous water reservoir, moderating soil moisture tension and enabling gradual release to plant roots, while also reducing nutrient leaching^15–17^. Fe-NB significantly increases soil water-holding capacity and aggregate stability, a critical function in moisture-limited environments^18,19^. Furthermore, by augmenting soil organic matter and stimulating beneficial microbial activity, Fe-NB enhances rhizosphere nutrient cycling under stress conditions^14,20^. Although not directly quantified herein, a plausible physiological mechanism involves the attenuation of oxidative stress. Prior evidence suggests such amendments can upregulate antioxidant enzyme systems (e.g., SOD, CAT, POD), scavenging reactive oxygen species (ROS) generated by combined HM and drought stress^21,22^. Our findings corroborate and extend the documented effects of soil amendments. The rise in soil pH following fine-grained Fe-SZ application is consistent with its base-cation release (e.g., Ca^2^_+_, K_+_) via exchange with H_+_ or NH₄_+_^23–25^. The concomitant improvements in soil organic carbon (OC), total nitrogen (TN), CEC, and available phosphorus (AP) align with the known roles of Fe-SZ and Fe-NB in nutrient retention and mineralization^26–29^. The pronounced positive response in wheat yield components to Fe-SZ mirrors results in other crops, where zeolites enhance nutrient- and water-use efficiency^17,30,31^. Similarly, the growth promotion by Fe-NB is well-supported, attributed to improved soil structure, nutrient supply, and the bioactivity of organic constituents^19,20,32^. The strong correlation between grain yield and soil OC/AP (explaining 91% and 83% of variability, respectively) powerfully reinforces the principle that restoring soil fertility is paramount for productivity in degraded arid and semi-arid agroecosystems^20,33^.

### Critical analysis of anomalous metal mobility

A salient and unexpected result was the increased bioavailability of certain metals with specific Fe-SZ treatments^33,34^. This paradox underscores the complexity of soil-amendment- contaminant interactions and challenges the assumption of universal immobilization^35^. Plausible explanations include: (i) the non-selective nature of ion exchange, where added cations from Fe-SZ (e.g., Ca^2^_+_) displace adsorbed HMs from soil colloids, transiently increasing their solubility^11^; (ii) amendment-induced shifts in pH or redox potential that alter metal speciation for specific elements; and (iii) the formation of soluble metal–organic or metallo-silicate complexes. This observation is critical, as it highlights the element- and context-specific nature of remediation strategies and underscores the necessity for tailored amendment protocols based on comprehensive soil and contaminant profiling^34^.

The combined application of stilbite zeolite and nanobiochar significantly improved wheat growth and biomass under drought and heavy metal stress, likely due to enhanced soil cation exchange capacity, water retention, and nutrient availability^36,37^. Zeolite has been reported to immobilize toxic metals, reducing their phytoavailability, while biochar enhances soil structure and stimulates beneficial microbial activity. These synergistic effects support plant physiological performance under stress conditions^9,11,34^. Biochar and nanobiochar amendments not only reduce heavy metal uptake but also modulate rhizosphere microbial communities, promoting stress resilience through improved nutrient cycling and antioxidant responses^38,39^. Similar observations have been reported for microbial-assisted phytoremediation systems, where inoculation with metal-tolerant fungi or PGPR strains enhanced plant growth and metal immobilization^12,21,24^. The capacity of microbes to degrade organic contaminants while facilitating metal stabilization further underscores the importance of integrated amendment-microbe strategies^33^.

Heavy metal stabilization was evident as treatments reduced As, Cd, Pb, and Ni accumulation in wheat tissues, consistent with previous studies showing mineral and organic amendments suppress metal mobility. Concurrently, amendments influenced greenhouse gas emissions, with reductions in N_2_O and CO_2_ fluxes reported in amended soils^40,41^. These findings highlight the potential for amendments to simultaneously improve soil quality and mitigate environmental risks^3,42^. The efficiency of soil amendments depends on particle size, application rate, and soil properties. Excessive application may disrupt nutrient cycling or microbial balance, while insufficient doses may be ineffective^4,10,43^. Long-term stability of immobilized metals is influenced by soil redox conditions, root exudates, and interactions with soil organic matter, which may remobilize metals under fluctuating environmental conditions^30,44^. Therefore, site-specific optimization and continuous monitoring are crucial for sustainable remediation^18^. Amendments also improve plant drought resilience by modulating water-use efficiency, osmolyte accumulation, and antioxidant enzyme activities^45,46^. These physiological enhancements complement chemical immobilization of metals, representing a holistic approach to stress mitigation^47–49^. Similar trends have been observed in maize and rice^50,51^, where integrated soil-microbe management enhanced growth under combined heat and drought stress^35^. The combination of stilbite zeolite and nanobiochar offers a technically robust strategy for enhancing wheat growth and phytostabilization under combined abiotic stresses^34^. Future studies should focus on field-scale validation, microbial community dynamics, and long-term ecological safety to ensure sustainable and scalable application.

### Future research trajectories

The insights from this controlled pot experiment must be interpreted within its inherent limitations. The use of a single, artificially contaminated soil type precludes broad generalization, as amendment efficacy is intrinsically linked to native soil properties such as mineralogy, pH, and initial organic matter content. The pot environment cannot replicate field-scale heterogeneity, including macroporosity, root architecture dynamics, and long-term climatic variability, all of which govern amendment longevity and performance. Moreover, discussions of microbial and molecular mechanisms including shifts in rhizosphere communities, hormonal signaling, and differential gene expression remain inferential. Direct empirical evidence from microbiological, transcriptomic, or proteomic analyses was beyond the present scope. Consequently, future research must prioritize long-term field validations across diverse pedoclimatic regimes to assess agronomic viability and economic feasibility. Integrated studies employing advanced spectroscopic, isotopic, and omics techniques are essential to deconvolute the precise biogeochemical and molecular interactions driving the observed benefits. Such a multi-faceted approach is indispensable for transforming these promising amendments into robust, scalable strategies for sustainable soil management and climate-resilient agriculture.

## Conclusions

The findings revealed the combined efficacy of Fe-SZ and Fe-NB treatments in alleviating both heavy metal contamination and drought stress, highlighting the critical importance of optimizing application rates and particle sizes to maximize remediation outcomes. Specifically, the application of 0.5 mm Fe-SZ and Fe-NB, each at 8% w/w, significantly reduced heavy metal concentrations while enhancing key soil fertility indicators, including organic carbon (OC), total nitrogen (TN), cation exchange capacity (CEC), and available phosphorus (AP). Notably, the synergistic use of native Fe-SZ activated by Fe-NB further improved soil nutritional status and reactivity, primarily through increased CEC, an essential determinant of water retention and nutrient availability under drought conditions. These enhancements translated into substantial gains in wheat productivity, particularly under conditions of water scarcity, low OC content, and alkaline pH, factors commonly limiting crop performance. In light of these results, further research into the long-term stability of these amendments and their mechanistic interactions with heavy metals and drought stress is warranted to support their broader application in sustainable soil management under challenging agro-environmental conditions.

## Data Availability

All data generated during this study are included in this published article and The datasets used during the current study available from the corresponding author on reasonable request.
